# A facile hydrothermal synthesis of high-efficient NiO nanocatalyst for preparation of 3,4-dihydropyrimidin-2(1*H*)-ones

**DOI:** 10.1038/s41598-022-12589-4

**Published:** 2022-05-20

**Authors:** Maryam Khashaei, Leila Kafi-Ahmadi, Shahin Khademinia, Ahmad Poursattar Marjani, Ehsan Nozad

**Affiliations:** 1 Department of Inorganic Chemistry, Faculty of Chemistry, Urmia University, Urmia, Iran; 2 Department of Inorganic Chemistry, Faculty of Chemistry, Semnan University, Semnan, Iran; 3 Department of Organic Chemistry, Faculty of Chemistry, Urmia University, Urmia, Iran

**Keywords:** Catalysis, Green chemistry

## Abstract

The present work introduces a one-step and facile hydrothermal procedure as a green process for the first time to synthesize nickel(II) oxide (NiO) nanoparticles. The as-prepared nanomaterials were used as high efficient, low toxic and cost catalyst for the synthesis of some organic compounds. Ni(NO_3_)_2_ and some natural extract were used as a surfactant for the first time to synthesis NiO nanomaterials. A high synthesis yield (91%) was obtained for S_2_. Rietveld analysis affirmed the cubic crystal system of the obtained NiO nanocatalyst. The morphology studies were carried out with the FESEM method and the images revealed a change from non-homogenous to homogenous spherical particles when the Barberryas was used instead of orange blossom surfactant. Besides, the images revealed that the particle size distribution was in the range of 20 to 60 nm. The synthesized catalysts were used for the first time in Biginelli multicomponent reactions (MCRs) for the preparation of 3,4-dihydropyrimidin-2(1*H*)-ones (DHPMs) under the present facile reaction conditions. High yield (97%) of the final product was achieved at the optimum condensation reaction conditions (Catalyst: 60 mg; temperature: 90 °C and time: 90 min) when ethyl acetoacetate/methyl acetoacetate (1 mmol), benzaldehyde (1 mmol) and urea (1.2 mmol) were used. A kinetic study affirmed pseudo-first-order model for Biginelli reactions followed the pseudo-first-order model.

## Introduction

Researchers are intensely interested in the synthesis of nanomaterials due to their specific properties. The nanomaterials functions are strongly dependent on their shape and size, making these factors highly considerable for their applications^1^. Nickel oxide (NiO) is a broad band gap antiferromagnetic p-type semiconductor^2^. NiO nanoparticles are used in gas sensors^3^, batteries^4^, transparent conducting layers^5^, solar thermal absorbers^6^, electrochromic devices^7^, optical fibers^8^, smart windows^9^ and photocatalysts^10^.

It has been reported that NiO has the optical band gap of 2.3–3.5 eV^11,12^. Various methods including co-precipitation^11^, sol–gel^12^, thermal decomposition^13^, microwave pyrolysis^14^, solvothermal^15^, anodic arc plasma^16^, sonochemical^17,18^, microemulsion^19^, hydrothermal^20^, solid-state^21^, boiling^22^ methods, green biosynthesis^23^ and etc. have been reported for NiO synthesis. Green synthesis is a field of study aiming to design clean products and sustainable processes by reducing or even not using the unsafe solvents nor toxic reagents. In this respect, the green synthesis is potential for environmental sustainability promotion. In addition, ecofriendly synthesis reactions fulfill the green chemistry essentials that lead to preparation of low cost, straightforward, stable, and relatively reproducible nanomaterials^24–30^.

Plant extracts, fungi, enzymes, and microorganisms are used as raw materials for green synthesis of nanoparticles. A plant-extract controlled nanoparticle synthesis has received a lot of attention due to its versatility^31,32^.

Application of plant extracts as reducing, stabilizing, and capping agent accompanied by other notable features such as ease of use, cost effectiveness, and environmental friendliness make them highly valuable materials^30,33^.

The capped nanoparticles can be prepared using plant extract phytochemicals as strong reducing agents that decrease the number of process steps, the cost and chemical use as a consequence^31,32,34^.

In an aqueous system, phytocompounds are substituted with chemicals and organic/inorganic solvents^35^. Extract of plant’s different parts like flower, leaf, fruit and etc. are currently used to prepare nanoparticles^32,36,37^.

**Figure 1:**
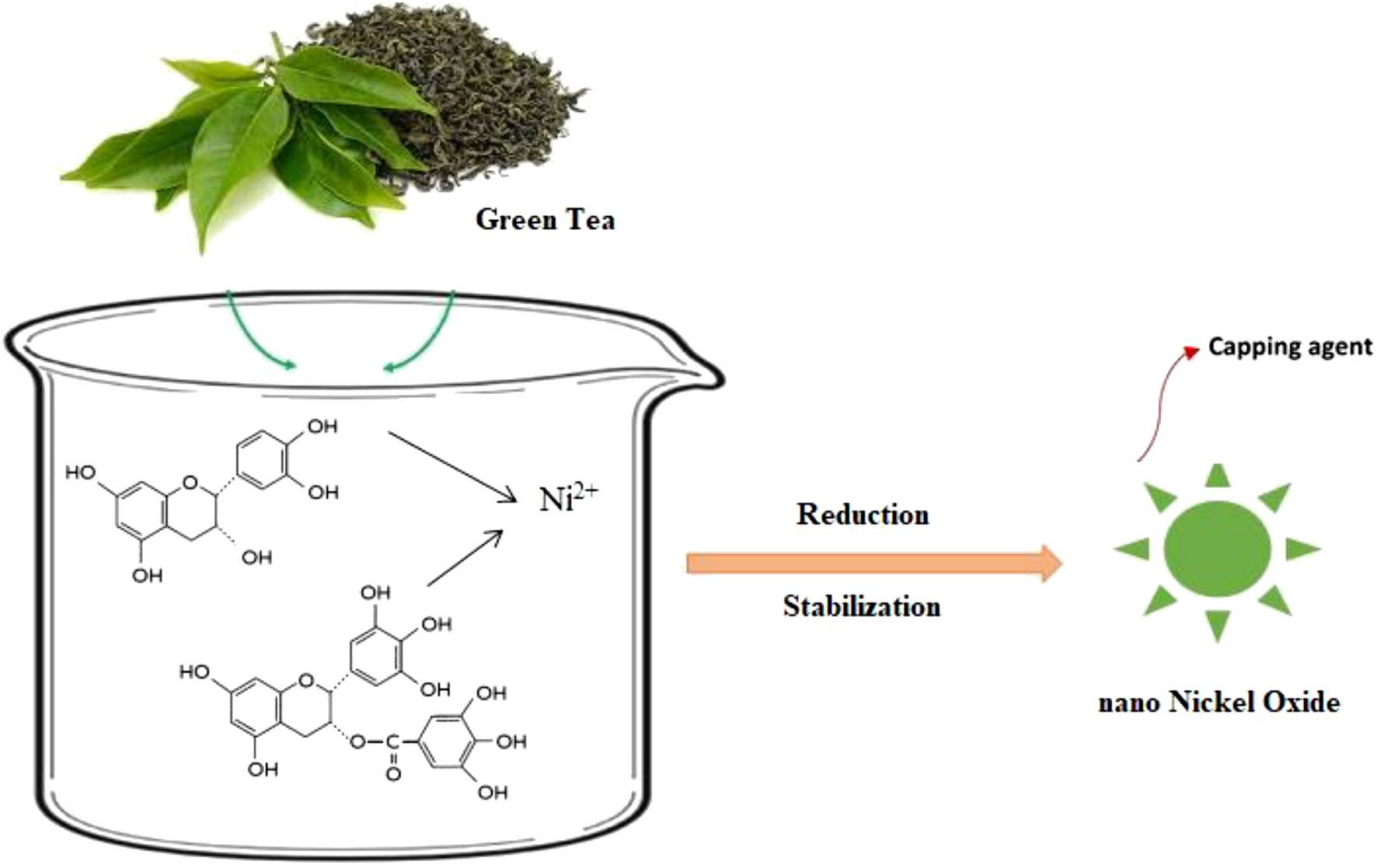
Schematic representation of the biosynthetic pathway of NiO nanoparticles using green tea extract for example.

The active biomolecules consist of amino, hydroxyl, carboxyl bifunctional groups, alkaloids, flavonoids, and terpenoids that would act as the reductant of metal salts and protective agents to form stabilizing layer on the biosynthesized nanoparticles (Fig. 1)^23,25,26,29–32,38^.

Multicomponent reactions (MCRs) are some reactions that three or more reagents react in a one-pot process to form a new product. MCRs have obtained attentiveness in synthesis and medicinal chemistry in as much as the advantage of one-pot single reaction preparation of some strategic organic molecules and structures^39,40^. Such reactions not only reduce the energy and solvent required but also lead to highly selective products. Owing to multiple benefits of MCRs process, the development of novel eco-friendly and NP-catalyzed MCRs procedures have been considered as an interesting growing field of research in organic chemistry^27,28,41,42^.

Biginelli reaction is a procedure for the synthesis of 3,4-dihydropyrimidin-2(1*H*)-ones (DHPMs) in a one-step process. These compounds have wide biological antihypertensive, antiviral, antitumor, antibacterial, antioxidant and anti-inflammatory applications^43,44^.

Several researches have confirmed the biological activities of DHPMs and their successful synthesis through Biginelli reactions via different metal oxides have been reported^45–48^.

**Figure 2:**
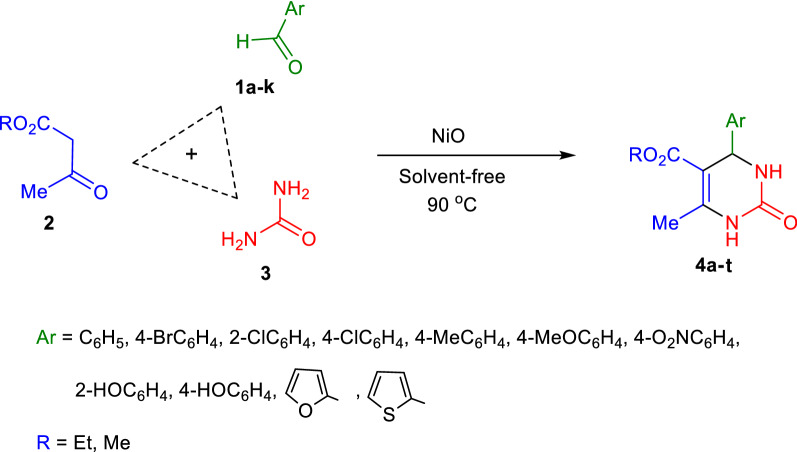
Schematic representation of the reaction pathway for the synthesis of DHPMs.

As part of our ongoing attempts in the field of catalysts^48,49^ to find a reusable high yield catalyst with low cost and toxicity, NiO nanocatalyst was synthesized by a novel and simple surfactant assisted mild condition hydrothermal procedure. Hibiscus tea, Orange blossom, Yarrow, Green tea, Barberries and Rosemary were used as natural surfactants in order to explore their effects on the growth of crystals, morphology and catalytic performance of NiO semiconductors. Rietveld analysis data were applied to investigate the extract effect on the experimental crystallographic properties of the prepared nanocatalyst. The optical properties and morphology of the materials were studied by UV–Vis spectroscopy and FE-SEM respectively. The high catalytic performance of the synthesized NiO nanomaterials was confirmed by their application as novel heterogeneous catalysts for the synthesis of DHPMs by Biginelli MCRs (Fig. 2). Besides, optimization of the factors (amount of catalyst, time and temperature of reaction) affecting the DHPMs synthesis process was evaluated by experimental design method. Therefore, synthesis of highly pure NiO nanoparticles by a new route and its application as a novel, low toxic, inexpensive, and high efficient catalyst to synthesize DHPMs is reported in this research for the first time.

## Experimental

### Materials and instruments

All precursors for the synthesis were purchased from Merck Co. (Germany) and no excess purification was carried out. Analytical grade of Ni(NO_3_)_2_·6H_2_O and 25 V/V% NH_3_ were prepared from Merck Co. The Hibiscus Sabdariffa L. Tea (Hibiscus tea), Citrus Sinensis (Orange blossom), Achillea Millefolium (Yarrow), Camellia Sinensis (Green tea), Barberis (Barberries) and Salvia Rosmarinus (Rosemary) were purchased from local market, Urmia, West Azerbaijan, Iran and all permissions were obtained for their application. The plants were then processed to obtain the desired extracts. X-ray Powder Diffraction (PXRD) patterns were recorded by a D5000 powder X-ray diffractometer (Siemens AG, Germany) using CuKα radiation to make phase identification. FullProf software was applied to accomplish the Rietveld analysis. Lattice parameter (a), sinθ/λ, FWHM (B(rad)), Bragg residual factor (RBragg), residual factor (RF), profile residual factor (Rp), weighted profile residual factor (Rwp), expected residual factor (Rexp), the goodness of Rietveld refinement (χ^2^), growth, purity values and crystal phase type, were investigated by Rietveld analysis. The FE-SEM images were taken on a Hitachi model S-4160 for morphologic studies. Absorption spectra were obtained with a UV–visible spectrophotometer (UV-1650 PC, Japan). A Tensor 27 FTIR spectrometer (Bruker, Germany) was used to obtain the spectra. Thin layer chromatography (TLC) using ethyl acetate/n-hexane mixture was used to evaluate the products purity. The melting points of the as-prepared DHPMs were measured using thermo scientific 9100 apparatus. ^1^H NMR spectra were recorded by a Bruker Avance DRX-400 spectrometer (Bruker, Germany) using TMS as internal reference and in DMSO-*d*_*6*_ as solvent.

### Plant extraction process

All plants were dried and grounded to obtain a powder. 300 mg was poured in 30 mL of distilled water under agitation at raised temperature up to 70 °C for a short period. The final extracts then were cooled down following by filtration process and kept at a decreased temperature (4 °C) until next usage. This process was repeated for all plants.

All experimental protocols were performed in accordance with relevant institutional, national, and international guidelines and legislation.

### Green synthesis of NiO nanomaterials

In a typical experiment, Ni(NO_3_)_2_·6H_2_O (7 g) was dissolved in deionized water (50 mL). Then, after adding one of the natural surfactants (20 mL) [Orange blossom (S_1_), Yarrow (S_2_), Hibiscus tea (S_3_), Green tea (S_4_), Rosemary (S_5_) and/or Barberry (S_6_)], the pH was adjusted at 7 using a 25 V/V% ammonia solution and then stirred for 48 h on a magnetic stirrer. In the next step, the obtained solution was kept under ultra-sonication (30 min) and the final solution was poured into an autoclave, sealed and heated up to 80 °C for 24 h. Then, the reaction container was cooled down by water immediately and the synthesized nanocatalyst was washed with distilled water and dried at 115 °C for 2 h. To obtain the final product, the nanocatalyst was treated in an electrical furnace at 410 °C for 10 h to remove the residual organic components and a gray-black powder was obtained.

### Synthesis of DHPMs

A mixture of arylaldehyde (1 mmol), ethyl acetoacetate/methyl acetoacetate (1 mmol), urea (1.2 mmol) and 30 mg (0.04 mol%) of the nanocatalyst were mixed and stirred. Biginelli reaction parameters were optimized with design expert software. After reaction completion, the final solid crude product was rinsed with deionized water to remove the unreacted materials. The catalyst was separated from the precipitated solid by dissolving the compound in ethanol. Then, the filtrated solution was left undisturbed to obtain the pure product crystals. In the experimental design of the reactions, the effective parameters were changed simultaneously until an optimum condition was obtained to find out the best reaction yield. The yield of each reaction was determined with mmol fraction measurement of the considered DHPMs. The spectra data of the prepared compounds are provided in supporting information.

## Results and discussions

### Characterization

#### X-ray diffraction analysis

**Figure 3:**
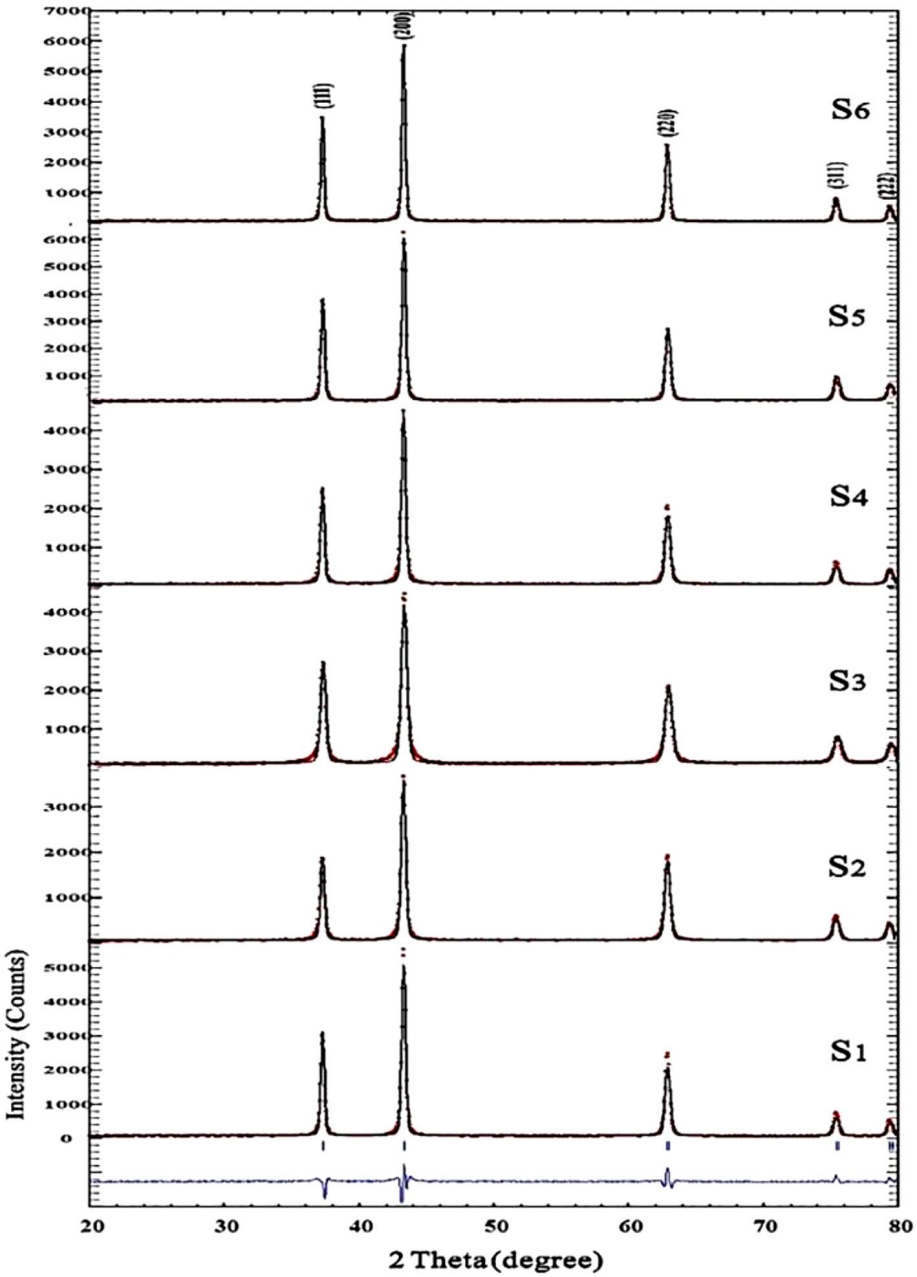
PXRD patterns of the NiO nanomaterials synthesized by hydrothermal method at 80 °C for 24 h followed by a calcination at 400 °C for 10 h refined by the Rietveld analysis.

The as-synthesized NiO samples were characterized by XRPD patterns (Fig. 3). To study the structural properties of the synthesized nanomaterials, the XRD data were analyzed by *FullProf* program. In the Rietveld analysis accomplished by the *Fullprof* program, the observed and calculated data are shown in red and black lines respectively that are obtained with the Rietveld refinement. The blue line is the difference: Yobs-Ycalc. The patterns indicate a pure NiO cubic crystal structure with Fm-3 m space group for S_1_ to S_6_ nanocatalysts^2–6^. The peaks positions emerging at 2θ = 37.23°, 43.26°, 62.83°, 75.36° and 79.35° are indexed to (111), (200), (220), (311), and (222) crystal planes of the as-synthesized NiO nanomaterials, respectively. The above-mentioned characteristic diffraction peaks can be perfectly indexed to the face-centered cubic (FCC) crystalline structure in view of their peak position and relative intensity. It can be observed that the samples are single phase and no peak related to impurity was observed except the characteristic peaks of FCC phase. According to Table 1, changing the surfactant type has no considerable effect on the unit cell volume of the targets. However, it was found that the crystal phase growth is varied by changing the surfactant type. The lowest and highest crystal phase growth is achieved when Yarrow and Barberry surfactants are used respectively.

**Table 1:** Synthesized nanocatalysts Rietveld analysis and unit cell parameters.

Entry	Sample	a	V	R_F_	R_B_	χ^2^	R_p_	R_wp_	R_exp_	Counts	Synthesis yield (%)
**1**	S_1_	4.18	73	0.81	1.24	3.8	20	20	10	5613	85
**2**	S_2_	4.18	73	0.54	0.86	4.16	23	24	12	3744	91
**3**	S_3_	4.17	73	0.51	0.80	7.90	30	28	10	4543	88
**4**	S_4_	4.18	73	0.69	1.1	4.23	25	24	11	4563	88
**5**	S_5_	4.18	73	0.67	1.00	3.78	19	19	10	6319	80
**6**	S_6_	4.18	73	0.87	1.32	2.64	17	18	11	6336	82

Some crystallographic parameters such as crystallite size (D), strain (ε), interplanar spacing (d), dislocation density (δ), and X-ray density (D_x_) were calculated and tabulated in Table 2. The crystallite size data were calculated by Scherrer equation:

1 $$D = \frac{K\lambda }{{B_{\frac{1}{2}} \cos \theta }}$$

**Table 2:** Crystallite size (D), dislocation density (δ), strain data (ε), interplanar spacing (d) and X-ray density (ρ_x_) of the as-synthesized nanomaterials.

Entry	Sample	2θ	B (rad)	D (nm)	δ	ε	d_Bragg_ (Å)	d_hkl_ (Å)	ρ_x_ (g/cm^3^)	SSA (cm^2^/g)
**1**	S_1_	43.26	0.006	25	0.0017	1.41	2.08	2.09	6.2	35
**2**	S_2_	43.25	0.007	22	0.0021	1.60	2.08	2.09	6.2	40
**3**	S_3_	43.31	0.007	21	0.0023	1.66	2.08	2.09	6.2	42
**4**	S_4_	43.26	0.006	24	0.0017	1.44	2.08	2.09	6.2	40
**5**	S_5_	43.28	0.006	25	0.0016	1.36	2.08	2.09	6.2	35
**6**	S_6_	43.26	0.005	30	0.0011	1.15	2.08	2.09	6.2	32

According to Table 2, crystallite size changes by surfactant type. In this respect, the smallest and the largest crystallite size values are obtained when Hibiscus tea and Barberry surfactants are used respectively.

The d values calculated by Bragg equation and Eq. (2) are in high consistency.

2 $$\frac{1}{{d^{2} }} = \left( {\frac{{h^{2} + k^{2} + l^{2} }}{{a^{2} }}} \right)$$

The dislocation parameter is a defect in a crystal related to the lattice improper resigestration of crystals from one part to another part. Hence, the defects in crystal system can be found using this parameter. The dislocation density δ [(lines/m^2^)10^14^] is calculated by the crystallite size (D) values with Eq. (3):

3 $$\delta = \frac{1}{{D^{2} }}$$

Change in dislocation density by varying the surfactant type was deduced. However, the change is not considerable. The highest and lowest δ values were obtained when Hibiscus tea (S_3_) and Barberry (S_6_) surfactants were used respectively.

The parameter strain ε (10^–3^) value was determined with Eq. (4):

4 $$\varepsilon = \frac{{\beta_{hkl} \cos \theta }}{4}$$

According to Table 2, increase in the strain value with changing the surfactant type ion is probably because of the change in the crystallite degree. The smallest ε value is obtained when Barberry (S_6_) is used.

The parameter X-ray density (ρ_x_) can be calculated by the following equation (Table 2):

5 $$\rho_{x} = \frac{ZM}{{Na^{3} }}$$

where M is the molecular weight of NiO (MW = 74.7 gmol^−1^), N is the Avogadro number, Z is the number of formula unit per unit cell for NiO (Z = 4) and *a* is lattice parameter. As it is observed from ρ_x_ data, it is found that the X-ray density value is low and its variation by surfactant change is not notable. It can be concluded that no considerable change is occurred due to the impurity intercalation (with different density and atomic weight), when surfactant type is changed.

The specific surface area (SSA) per unit volume is a characteristic that can affect physical properties, chemical reactivity and photochemical efficiency. SSA can be calculated by measuring XRD density (ρ_xrd_) and mean particle size (D) according to the Eq. (6):

6 $$SSA = \frac{6000}{{D\rho_{xrd} }}$$

#### Morphology analysis

**Figure 4:**
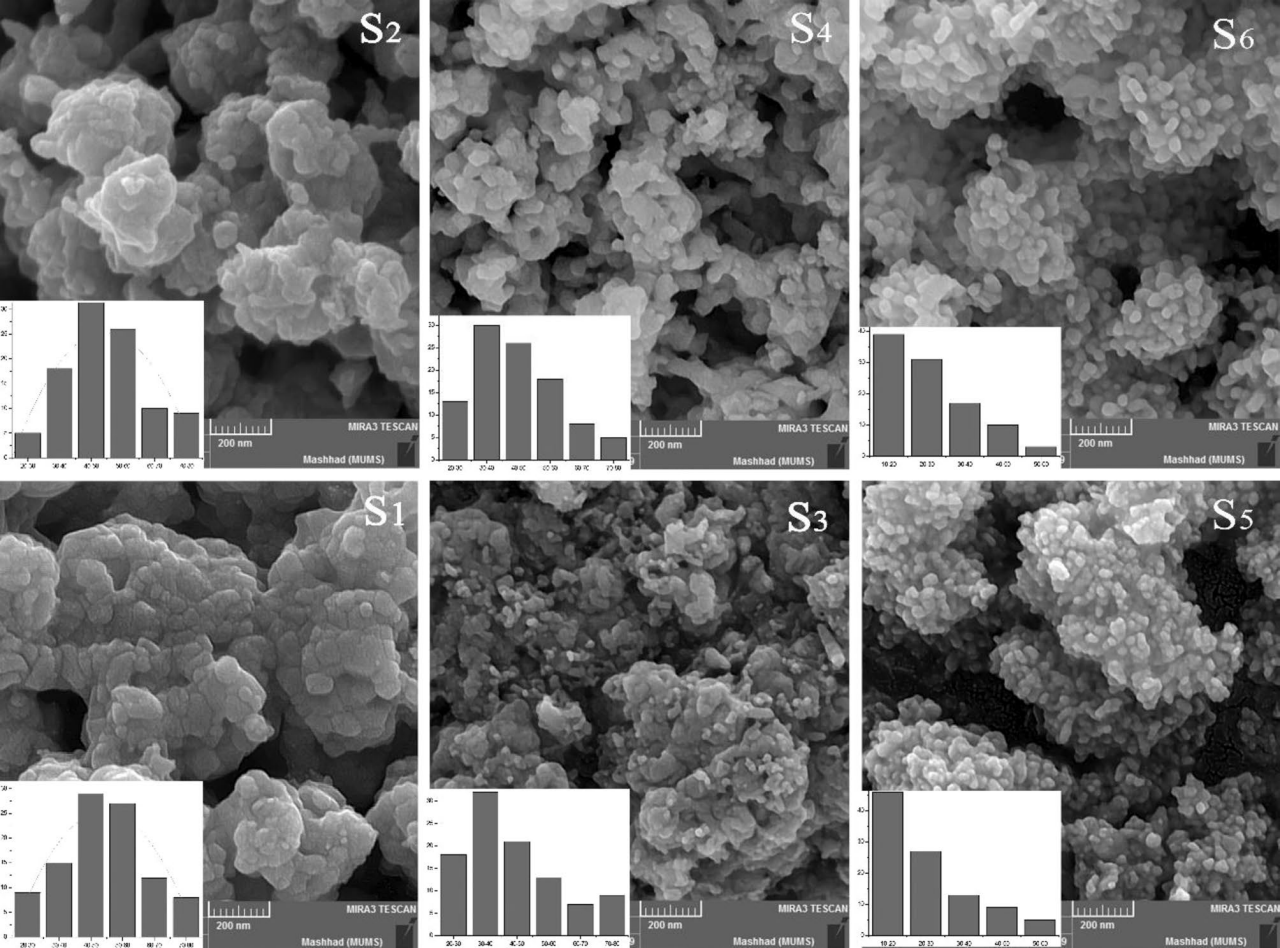
Effect of natural essence type on the morphology and the particle size distribution of synthesized NiO nanomaterials.

Figure 4 shows the FE-SEM images of the as-synthesized NiO nanomaterials. The insets are the particle size distribution calculated by software. The X and Y-axes are particle size (nm) and counts (%), respectively. As can be seen, the nanomaterials have porous morphology. It is clear that the final structure is formed by joining individual particles to each other. Besides, the size homogeneity and morphology of the particles are changed by surfactant type. In addition, no considerable homogeneity is observed for S_1_ to S_3_ but when Green tea (S_4_), Rosemary (S_5_) and Barberry (S_6_) are used, the homogeneity is enhanced while the particle size is decreased. It was observed that the particle sizes were about 40–60, 40–60, 30–40, 30–40, 20–30 and 20–30 nm, for S_1_–S_6_, respectively.

#### Optical properties

The physical properties of the as-synthesized nanomaterials were investigated by studying the absorption spectra and direct optical band gap energies and investigating the essence type effect on the parameters.

**Figure 5:**
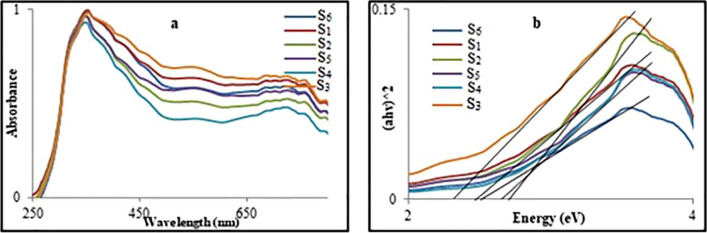
UV–Vis spectra (**a**) and NiO nanocatalyst band gap energy (**b**).

The UV–Vis are shown in Fig. 5a and b. According to the results of Pascual et al.^31^ (αhν)^n^ = A (hν − E_g_) is the relation between the absorption coefficient and incident photon energy, where A is constant and E_g_ is the direct band gap energy if n = 2. With extrapolating the linear part of the curve to the energy axis, the value of the direct band gap energies was evaluated. The E_g_ values were 2.45, 2.70, 2.30, 2.65, 2.65, and 2.50 eV, for S_1_–S_6_, respectively^34–36^. The data indicate that the essence type can affect the E_g_ that may be due to the effect on the crystallite size of the as-synthesized nanomaterials.

#### FT-IR spectroscopy

**Figure 6:**
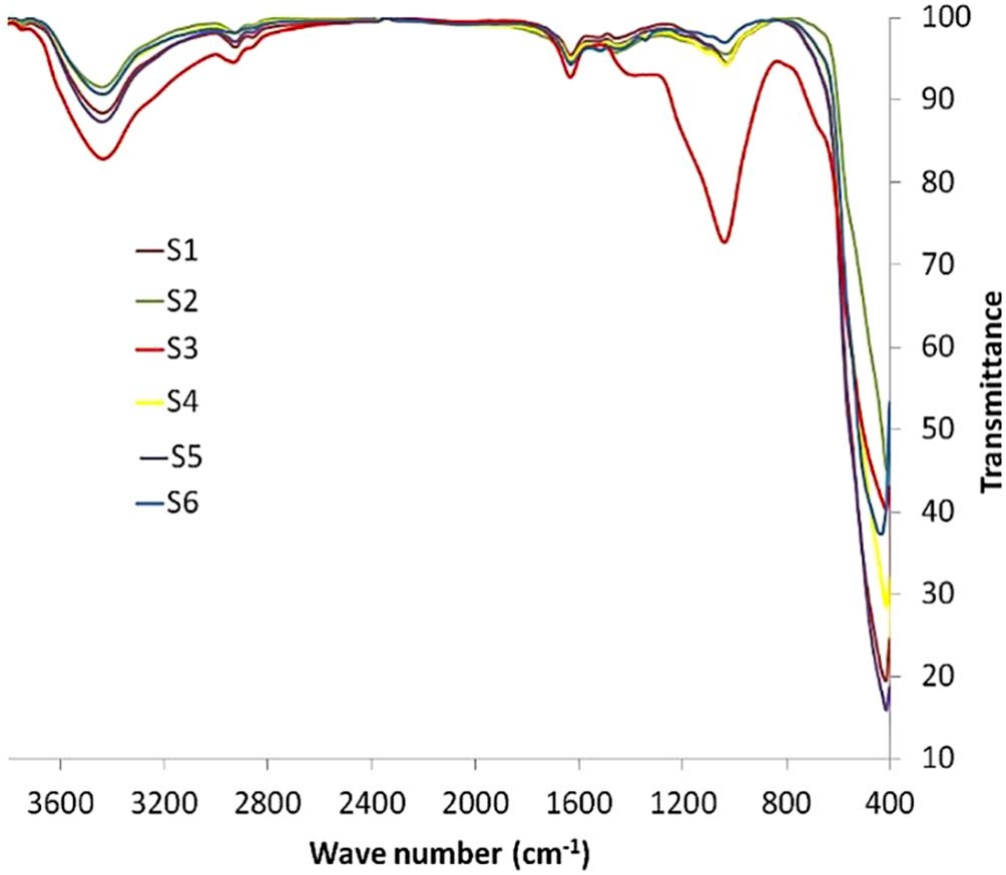
FTIR spectra of the prepared nanomaterials.

According to the FTIR spectra (Fig. 6), the absorption peaks are observed at 401–420, 617, 1018, 1620 and 3800 cm^−1^. The strong band at 401–420 and 617 cm^−1^ correspond to vibrations of Ni–O bonds^36,37^. The band at 1018 cm^−1^ is assigned to C–O stretching vibration that can be associated to the residual extract compounds^36^. The band at 1620 cm^−1^ can be attributed to the bending vibrations of water molecules. The peak at 3800 cm^−1^ is due to the O–H stretching vibrations^45,46^.

### Biginelli catalytic process

#### Achieving optimal conditions by response surface methodology

Several experimental designs can be used to find out the optimum level of factors in different reactions such as catalytic processes. Full factorial design is one of the most interested experiment designs that combine all possible combination of the factors and their settings. Response surface methodology (RSM) is one of the methods that analyzes the experimental design by mathematical and statistical method using an empirical model and to evaluate the model, analysis of variance (ANOVA) was used. A proper analysis of variance requires some repeated experiments^50^. In the present Biginelli reaction study, determination of the nanocatalyst amount, temperature and time was investigated and the outcome response is the yield (%). Various experiment designs of the aforementioned factors are tabulated in Table 3. The high and low factor levels are coded as (+ 1) and (− 1) respectively (Table 4). Four replicates at the center of the factors are considered for the validation of the model by ANOVA (Table 5). The factorial design data are fitted to a linear response model. Reaction yield (Y%) correlation with factors based on first order model is presented in Eq. (7):

7 $$Y\% = 60.80 + 15.63 \times A + 3.67 \times B + 5.05 \times C + 2.00 \times A \times B + 2.75 \times A \times C + 2.27 \times B^{2} + 2.27 \times C^{2}$$

**Table 3:** Three-level full factorial design.

Entry	Catalyst (mg)	Temperature (°C)	Time (min)	Yield (%)
**1**	30	60	90	49
**2**	60	60	60	69
**3**	45	75	75	62
**4**	60	90	90	94
**5**	45	75	75	61
**6**	45	75	75	61
**7**	45	100	75	73
**8**	30	90	90	53
**9**	45	75	75	62
**10**	30	60	60	47
**11**	45	75	75	60
**12**	45	50	75	61
**13**	60	60	90	82
**14**	45	75	75	60
**15**	45	75	100	78
**16**	20	75	75	34
**17**	30	90	60	50
**18**	70	75	75	86
**19**	60	90	60	80
**20**	45	75	50	56

Benzaldehyde, ethyl acetoacetate and urea (molar ratio: 1:1:1.2).

**Table 4:** Actual and coded forms of the designed experiments with the factors in CCD.

Entry	Factor	Name	Units	Low actual	High actual	Low coded	High coded	Mean	Std. dev.
**1**	A	Catalyst	Numeric	30.00	60.00	− 1.000	1.000	45.000	12.395
**2**	B	Temperature	Numeric	60.00	90.00	− 1.000	1.000	75.000	12.395
**3**	C	Time	Numeric	60.00	90.00	− 1.000	1.000	75.000	12.395

**Table 5:** ANOVA output data for suggested first-order model.

Entry	Source	Sum of squares	df	Mean square	F value	p-value prob > F	
**1**	Model	4099.6	7	585.66	249.23	< 0.0001	Significant
**2**	A	3336.22	1	3336.22	1419.76	< 0.0001	
**3**	B	184.39	1	184.39	78.47	< 0.0001	
**4**	C	348.61	1	348.61	148.35	< 0.0001	
**5**	AB	32	1	32	13.62	0.0031	
**6**	AC	60.5	1	60.5	25.75	0.0003	
**7**	B^2^	75.17	1	75.17	31.99	0.0001	
**8**	C^2^	75.17	1	75.17	31.99	0.0001	
**9**	Residual	28.2	12	2.35			
**10**	Lack of fit	24.2	7	3.46	4.32	0.0632	Not significant
**11**	Pure error	4	5	0.8			
**12**	Cor. total	4127.8	19				

The influence of each parameter on the reaction yield is shown by its coefficient in the equation. The more the value means higher effect. It can be seen that the catalyst is more effective than the other factors, in addition, the effect of temperature and time are close to each other.

Table 4, actual and coded forms of the designed experiments. The variables [amount of catalyst (A), temperature (B) and time (C)] are given in coded form (− α, − 1, 0, + 1, + α).

Investigation of the optimum conditions for the present Biginelli reactions was studied by ANOVA (Table 5) and the differences between group means were compared. This ratio of F-distribution (F-value) is the difference between group means. According to Table 5, the p-value of the regression was > 0.05 indicating that the model was significant at a high confidence level (95%)^50^. Lack-of-fit test was used to confirm the adequacy of the fitted model. At 95% confidence level, the *p*-values for the lack-of-fits are < 0.05, which is not significant. Furthermore, ANOVA results strongly support the aforementioned discussion. In addition, the regression square (R^2^), adjusted (R^2^-adj) and predicted (R^2^-prd) regression values were applied to study the fitting quality of the linear model equation. It is evident that the R^2^ = 0.99 for the variation fitting indicates high correlation of the response and the independent factors. Besides, high R^2^-adj = 0.98 and R^2^-prd = 0.97 reveals great relevance of the suggested model.

Optimum conditions for DHPMs synthesis were found with RSM. Figures S1 and S2 presented in the supplementary file shows the 2D and 3D plots, dispersal residuals and predicted values versus the experimental production efficiency data, respectively. The data revealed that the optimum condensation conditions of reaction were 60 mg of catalyst, 90 °C and 90 min reaction temperature and time respectively. Investigating the reaction kinetic of the present Biginelli reactions is important part to investigate the effect of parameters on the reaction rate. The graphs in Fig. S2, exhibit a linear dependence at 0–90 min following a first-order kinetic model for the two samples. The equations and the reaction time plot for kinetic investigations are presented in Eqs. S1, S2 and Fig. S3 of supporting information.

Table 6, presents the catalytic activity data of S_1_ and S_6_ at the optimum condition. The purity of the as-prepared DHPMs compounds is investigated by measuring the melting points of the recrystallized DHPMs. The yield data indicate that S_6_ catalytic activity is better than S_1_ in almost all of the reactions. This can be due to the higher crystallinity (counts value), smaller particle size and higher surface area of S_6_ than S_1_.

**Table 6:** Catalytic performance of S_1_ and S_6_ at optimum conditions.

Entry	Ar	R	Yield^a^ (%) S_1_	Yield^a^ (%) S_6_	m.p.^b^ (°C) S_1_	
					Obsd	Lit
**1**	C_6_H_5_CHO	EtO	94	97	201–203	202–203^51^
**2**	4-BrC_6_H_4_CHO	EtO	91	94	212–214	213–215^52^
**3**	2-ClC_6_H_4_CHO	EtO	77	91	216–218	216–219^52^
**4**	4-ClC_6_H_4_CHO	EtO	90	93	210–212	213–215^52^
**5**	4-MeC_6_H_4_CHO	EtO	91	92	213–215	214–216^53^
**6**	4-MeOC_6_H_4_CHO	EtO	75	79	201–203	201–202^52^
**7**	4-O_2_NC_6_H_4_CHO	EtO	53	61	206–210	207–208^52^
**8**	2-HOC_6_H_4_CHO	EtO	74	78	213–214	214–215^54^
**9**	4-HOC_6_H_4_CHO	EtO	86	86	233–235	232–234^55^
**10**	C_6_H_5_CHO	MeO	92	93	203–205	206–209^53^
**11**	4-BrC_6_H_4_CHO	MeO	84	87	241–243	240–242^52^
**12**	2-ClC_6_H_4_CHO	MeO	78	86	228–230	226–229^56^
**13**	4-ClC_6_H_4_CHO	MeO	89	91	202–205	204–207^52^
**14**	4-MeC_6_H_4_CHO	MeO	90	91	201–203	202–204^53^
**15**	4-MeOC_6_H_4_CHO	MeO	75	77	189–191	190–193^53^
**16**	4-O_2_NC_6_H_4_CHO	MeO	69	72	232–234	233–236^53^
**17**	2-HOC_6_H_4_CHO	MeO	67	74	264–267	265–268^54^
**18**	4-HOC_6_H_4_CHO	MeO	90	91	240–243	241–242^51^
**19**		EtO	92	93	207–210	209–210^57^
**20**		EtO	90	91	214–216	215–217^57^

^a^Yield refers to isolated pure products.

^b^Products were characterized by comparison of melting points with the known products reported in the literature.

The turnover numbers (TON, moles of product per mole of catalyst) for the synthesis of DHPMs were calculated by the following equation^58^. The mmol amount of NiO was 0.8 at the optimized conditions.

8 $$Turnover\;Number\;(TON) = \frac{mmol\;of\;product}{{mmol\;of\;catalyst}}$$

Turnover frequency is obtained by the below relationship^59^. t_1/2_ is 360 min (4 run) and Ln2 = 0.7.

9 $$Turnover\;Frequency\;(TOF) = \frac{0.7\;TON}{{t_{\frac{1}{2}} }}$$

S_1_ and S_6_ catalytic efficiency (Y%) comparison is presented in Table 7. It can be seen that the S_6_ catalytic activity is better than S_1_. In addition, the considerable yields were achieved when benzaldehyde, ethyl acetate and urea were used as raw materials.

**Table 7:** S_1_ and S_6_ catalytic performance comparison.

Entry	Sample	Ar	R	Product (mmol)	TON	TOF
**1**	S_1_	C_6_H_5_CHO	EtO	0.94	1.175	0.002285
**2**	S_1_	C_6_H_5_CHO	MeO	0.92	1.15	0.002236
**3**	S_6_	C_6_H_5_CHO	EtO	0.97	1.2125	0.002358
**4**	S_6_	C_6_H_5_CHO	MeO	0.93	1.1625	0.00226

**Figure 7:**
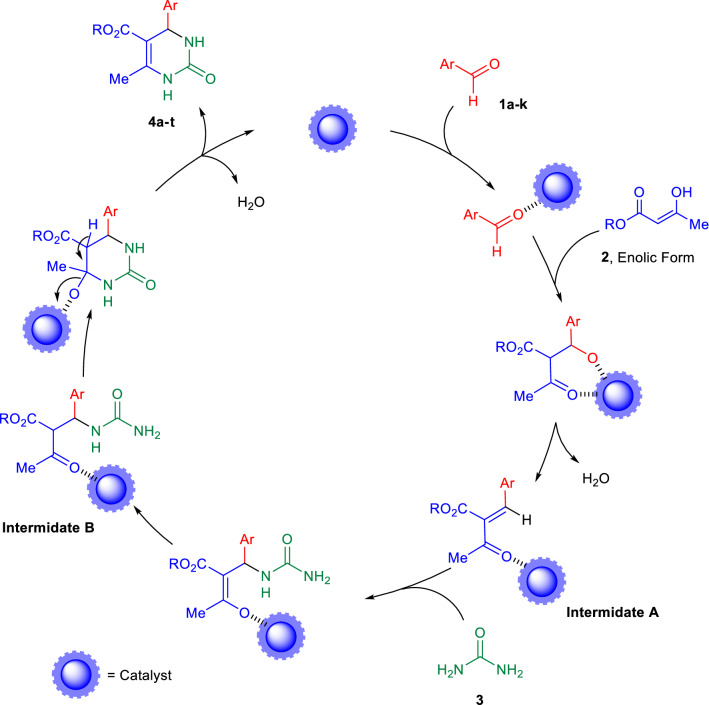
Proposed mechanistic route of MCRs Biginelli reaction catalyzed by NiO nanoparticles for the DHPMs synthesize.

The proposed reaction mechanism of the target products formation using NiO nanoparticles as catalyst is presented in Fig. 7.

In the initial step of reaction, the carbonyl groups of arylaldehydes **1a–k** and enolization of β-ketoester **2**, activated with nanoparticles occurs, afterwards, the *Knoevenagel* condensation between arylaldehydes **1a–k** with β-ketoester in enol-tautomeric forms to give the corresponding product as an intermediate α,β-unsaturated ketones **A** via dehydration.

In the next step, the *Michael* type addition of activated urea (**3**) molecules to the intermediate **A**, leads to the generation of open-chain ureide intermediate **B**, which, subsequently undergoes intramolecular cyclization of intermediate **B**, via elimination of H_2_O molecule to afford the six membered heterocyclic compounds as the target DHPMs **4a–t**. In the present work, it is worthwhile to mention the crucial role of NiO as a catalyst under solvent-free conditions in the remarkable accelerating of all reaction stages. The NiO nanoparticle facilitates the formation of target products through Lewis acid sites (Ni^+2^) coordinated to the oxygen of carbonyl groups. On the other hand, deprotonation of the C–H bond occurs in the presence of Lewis basic sites (O^−2^). As a result, the formation of DHPM derivatives proceeds by activation of reactants through both Lewis acids and basic sites of NiO nanoparticles.

**Figure 8:**
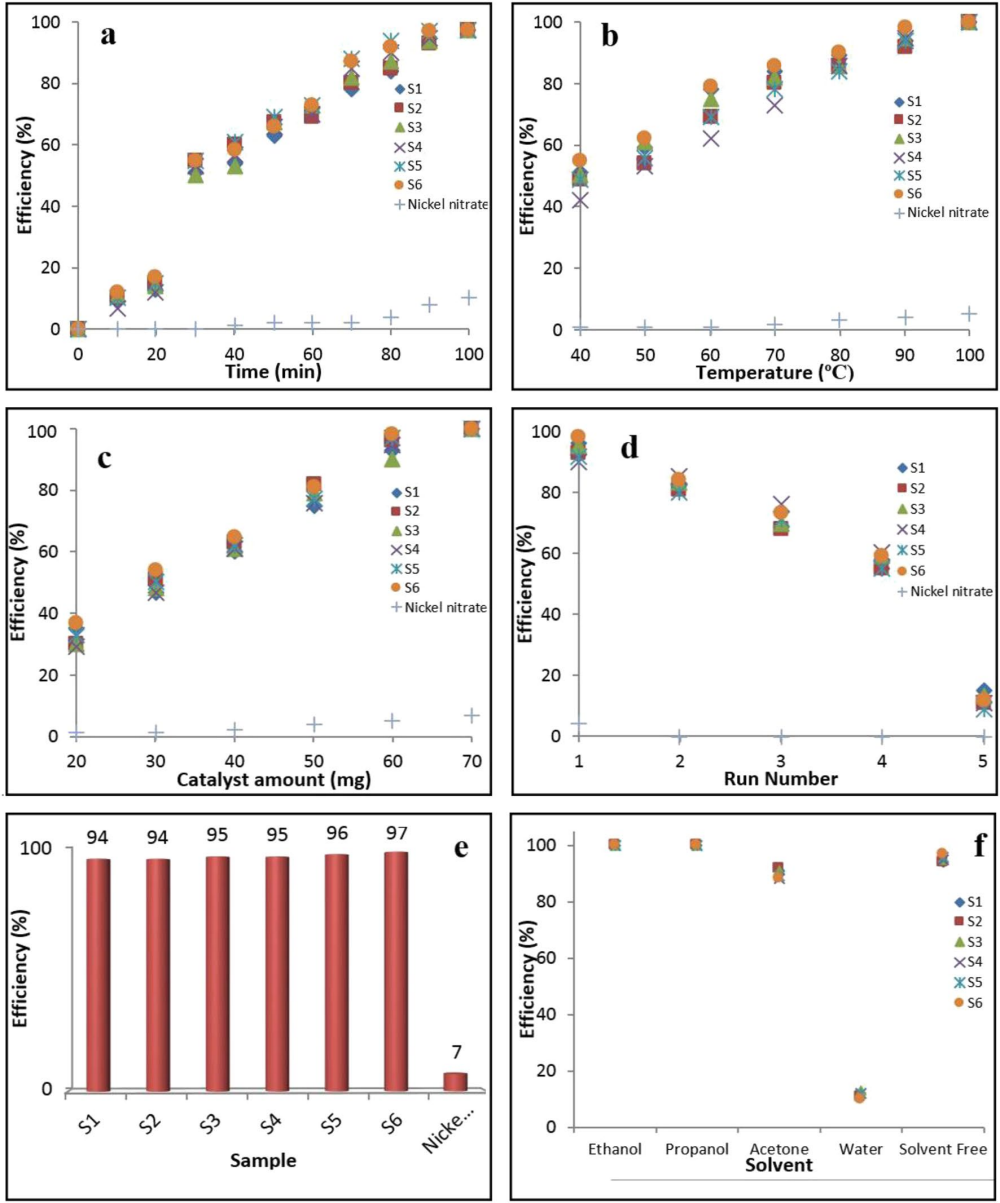
Effects of reaction time (**a**), temperature (**b**), catalyst amount (**c**), reusability (**d**), synthesized and raw material samples (**e**), and solvent type on the Biginelli catalytic efficiency (**f**). The experiments presented in (**d**–**f**), were done at the optimized conditions. The other experiments shown in the Figure done with the two factors at the optimized conditions.

In order to study the effect of a parameter on the Biginelli reaction yield, the synthesized catalysts activity is scrutinized in Fig. 8a–f. In the present study, when a parameter is changed (for example time), the other two parameters maintain at the optimum values (for example temperature and catalyst) (Fig. 8a–c). According to the data, the optimum values are 60 mg catalyst, 90 °C temperature and 90 min time in which the catalytic reaction yield is 97%. The experiments shown in Fig. 8d–f, are conducted at the optimized reaction conditions. The data reveal that increasing each parameter has a positive influence on the catalytic efficiency of the as-synthesized samples. In addition, the influence of reaction time and temperature are more than catalyst amount effect on the reaction yield. Figure 8d, reveals that the recyclability of all of the catalysts is up to run 4. As it can be seen from the Fig. 8e, it is clear that S_6_ keeps its activity longer than S_1_ in the same reaction conditions. Figure 8f, shows that the organic solvents added to the reaction mixture play a medium and solve the raw materials until the contact surface is increased and the efficiency is enhanced. However, when H_2_O was used as the solvent in the reaction mixture, it seems that it acts as an inhibitor and the reaction is not accomplished properly.

**Figure 9:**
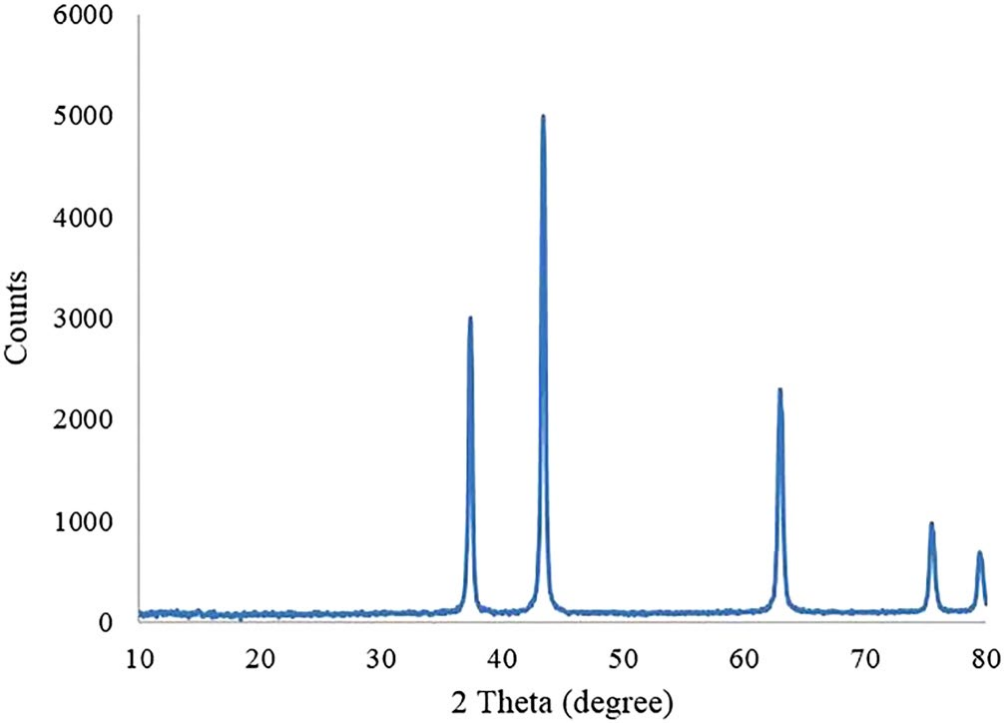
XRD pattern of S_5_ after 4 runs catalytic reaction.

Figure 9 presents the XRD pattern of NiO nanocatalyst after 4 cycles of catalytic reaction. As can be found from the pattern, it is clear that the counts value of the sample decreases considerably. However, the nature of NiO is still maintained unchanged.

A comparison of S_1_ and S_6_ catalysts with other previously reported catalysts for the synthesis of DHPMs using the H, 2-Cl and 4-Cl derivatives is tabulated in Table 8 that confirm the competency of this research. The most important factors affecting the reaction outcome: catalytic reaction, the catalyst amount, reaction time and temperature comparison with other reported researches affirms the excellence of current work. According to the data, some higher efficiencies are achieved only when the reaction duration and temperature were higher than the present research. Furthermore, composite materials^59–65^ have decreased yield in compare of other studies.

**Table 8:** Comparison of synthesized NiO nanocatalyst with other catalysts.

Entry	Catalyst	Ar	Catalyst amount	Reaction condition	Yield (%)	Time (min)	Ref.
**1**	Zn(CH_3_SO_3_)_2_.4H_2_O	C_6_H_5_CHO	3 mmol	Ethanol, reflux	64	7 h	^60^
**2**	CuI	C_6_H_5_CHO	15 mol%	Solvent-free, 90 °C	85	60	^61^
		4-ClC_6_H_4_CHO			84	90	
**3**	ZrO_2_–Al_2_O_3_–Fe_3_O_4_	C_6_H_5_CHO	50 mg	Ethanol, reflux, 140 °C	82	300	^62^
		2-ClC_6_H_4_CHO			40		
		4-ClC_6_H_4_CHO			66		
**4**	ZnO	C_6_H_5_CHO	25 mol%	Solvent-free, 90 °C	92	50	^63^
		4-ClC_6_H_4_CHO			95		
**5**	SbCl_5_.SiO_2_	C_6_H_5_CHO	100 mg	Solvent-free, room temperature	95	12	^64^
		4-ClC_6_H_4_CHO			93	20	
**6**	PANI–FeCl_3_	C_6_H_5_CHO	0.24 mmol	CH_3_CN, reflux	83	24 h	^65^
		2-ClC_6_H_4_CHO			84		
		4-ClC_6_H_4_CHO			90		
**7**	Sm^3+^–Doped Bi_2_Mn_2_O_7_	C_6_H_5_CHO	14 mg	Solvent-free, 104 °C	82	66	^66^
		2-ClC_6_H_4_CHO			71		
		4-ClC_6_H_4_CHO			37		
**8**	S_1_	C_6_H_5_CHO	60 mg	Solvent-free, 90 °C	94	90	This work
		2-ClC_6_H_4_CHO			77		
		4-ClC_6_H_4_CHO			90		
**9**	S_6_	C_6_H_5_CHO	60 mg	Solvent-free, 90 °C	97	90	This work
		2-ClC_6_H_4_CHO			91		
		4-ClC_6_H_4_CHO			93		

## Conclusion

This research aimed to report a systematic study of the surfactant type influence on the physical properties of the as-fabricated NiO nanomaterials and using the materials as low cost and toxicity and high efficient recyclable catalysts for the synthesis of DHPMs. NiO nanomaterials were synthesized with a green procedure. The Rietveld analysis data revealed that the surfactant type changed the crystallographic parameters. It was found that the lowest and highest crystal phase growth was achieved when Yarrow and Barberry surfactants were used, respectively. FE-SEM images revealed a change in the morphology from non-homogeneous to homogeneous spherical particles after change in surfactant type from orange blossom to barberryas. DHPMs optimum synthesis conditions were catalyst: 60 mg, reaction temperature: 90 ºC and reaction time: 90 min. The purity of the DHPMs was investigated by recrystallizing and measuring the melting points. The yield at the optimized conditions was 94% and 97% for S_1_ and S_6_, respectively. Langmuir–Hinshelwood (L–H) kinetic model affirmed the pseudo-first order kinetic model of present Biginelli reactions. The K_app_ values confirmed the faster reaction using S_6_ in compare to S_1._ Better catalytic efficacy was obtained for S_6_.

## Supplementary Information

Supplementary Information.

## Data Availability

All data generated or analyzed during this study are included in this published article [and its supplementary information files].
