# Supplementary material for: A facile hydrothermal synthesis of high-efficient NiO nanocatalyst for preparation of 3,4-dihydropyrimidin-2(1H)-ones
Source: Sci Rep. 2022 May 20;12:8585. doi: 10.1038/s41598-022-12589-4 (PMC9122962; doi:10.1038/s41598-022-12589-4)
Supplement: Supplementary file 1 — Supplementary Information. [file 41598_2022_12589_MOESM1_ESM.pdf]

Supplementary Information

A facile hydrothermal synthesis of high-efficient NiO nanocatalyst for preparation of 3,4-dihydropyrimidin-2(1*H*)-ones

Maryam Khashaei[1], Leila Kafi-Ahmadi[1,*], Shahin Khademinia[2], Ahmad Poursattar Marjani[3] & Ehsan Nozad[3]

[1]*Department of Inorganic Chemistry, Faculty of Chemistry, Urmia University, Urmia, Iran.*

[2]*Department of Inorganic Chemistry, Faculty of Chemistry, Semnan University, Semnan, Iran.*

[3]*Department of Organic Chemistry, Faculty of Chemistry, Urmia University, Urmia, Iran.*

*E-mail: l.kafiahmadi@urmia.ac.ir

The response surface methodology (RSM) was used to investigate the influence of the three factors (catalyst, time, temperature) on the present Biginelli reaction. 2D and 3D plots of the AB and AC interactions are exhibited in Figure S1. Interaction between variables is obvious due to the curvature of the plots. In other words, by increasing the reaction time at a constant reaction temperature (AC interaction), and by increasing the reaction temperature at a constant reaction time (AB interaction), high surface area of catalyst is available for the raw materials molecules leading to enhance the DHPM derivatives production percentage.

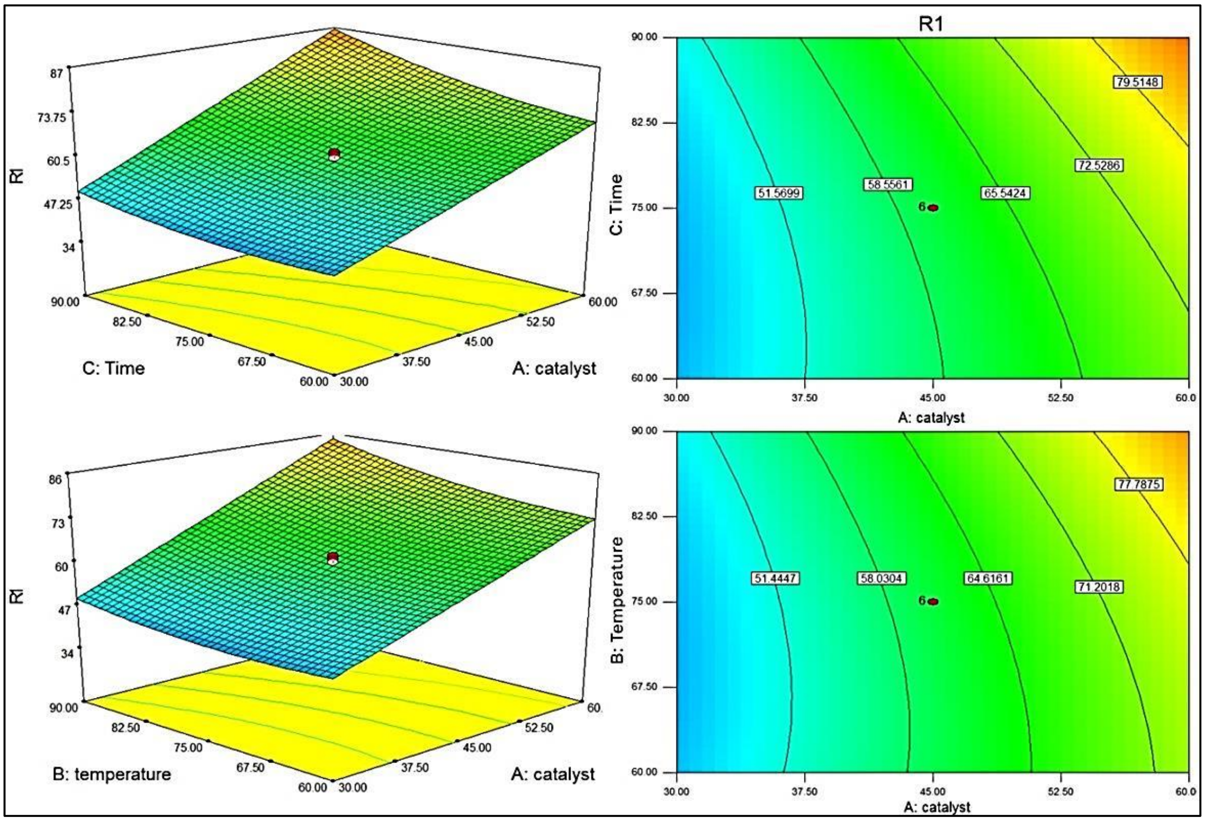

FIGURE S1 DHPMs synthesis 2D and 3D surface plots

Figure S2 shows the dispersal residuals and predicted values versus the experimental efficiency. A proper agreement between the predicted and experimental efficiency (R^2 = 0.99) is obtained. Besides, the data represent the adequacy and significance of the model and normal plots of the residual versus the studentized residuals as well. As it is evident, no significant dispersal is demonstrated because of appearing the data on a straight trend line. As could be seen from the Figure, it is clear that the Lambda value is near 1 showing the good optimization for obtaining the optimum conditions. Besides, Figure S2, shows the optimum values of the parameters obtained by the mentioned model. According to the obtained results, the optimum values for reaction time, catalyst amount and temperature are 90 min, 60 mg and 90 ºC, respectively.

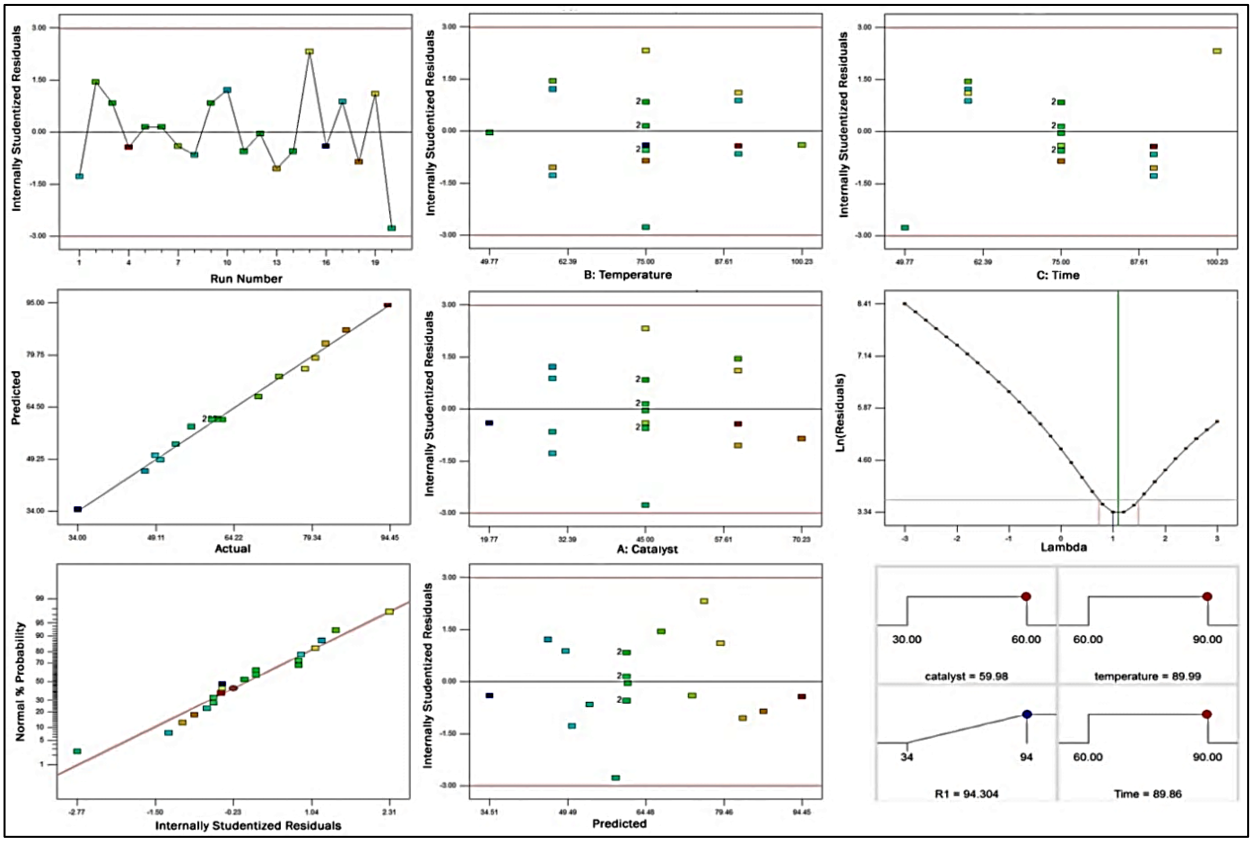

FIGURE S2 Plots of dispersal and normalized residuals, lambda and the optimized values for the Biginelli reaction

Scheme 1, shows a summary of the present Biginelli reaction pathway. As we could find from the optimization results obtained by design expert software (Figures S1 and S2), it was revealed that 60 mg of the catalyst, 90 ºC reaction temperature, and 90 min reaction time were the optimum parameters for the synthesis of DHPMs. The optimized parameters were used for the synthesis of other DHPM derivatives.

Investigation of the Biginelli reaction kinetic order

Finding the reaction kinetic of the present Biginelli reactions is important part to study the parameters effect on the reaction rate. Most of the catalytic processes kinetic order using heterogeneous catalyst are investigated through Langmuir–Hinshelwood (L–H) kinetic model (Eq. (S1)).

$$r_0 = -(dC_0)/dt = (kKC_0)/(1+kC_0) \quad \text{(S1)}$$

In which k, K_{app}, R^2 and r_0 are the adsorption coefficient of the reaction, the apparent rate constant of the reaction, correlation coefficient and the initial rate of the reaction respectively. The K_{app} and R^2 of the Biginelli reaction were studied by first-order kinetic model (Eq. (S2).

$$\ln[C_0]/[C] = K_{app}t \qquad \text{(S2)}$$

C_0 and C are the initial and at time t concentrations of ethyl acetoacetate and k is the first-order rate constant.

The data representing the catalytic action of S_1 and S_6 in Figure S3 affirm the first-order kinetic model due to the linear dependence at the time range of 0 to 90 min. The slope of the line represents the apparent rate constant (K_{app}). The amount of K_{app} and R^2 values are 0.0307 min^{-1} and 0.989 for S_1 and 0.0375 min^{-1} and 0.984 for S_6 showing the higher catalytic reaction of $S_{6.}$ This can be concluded from FESEM images that it is due to the smaller size and so, higher surface area of S_6 compared to S_1.

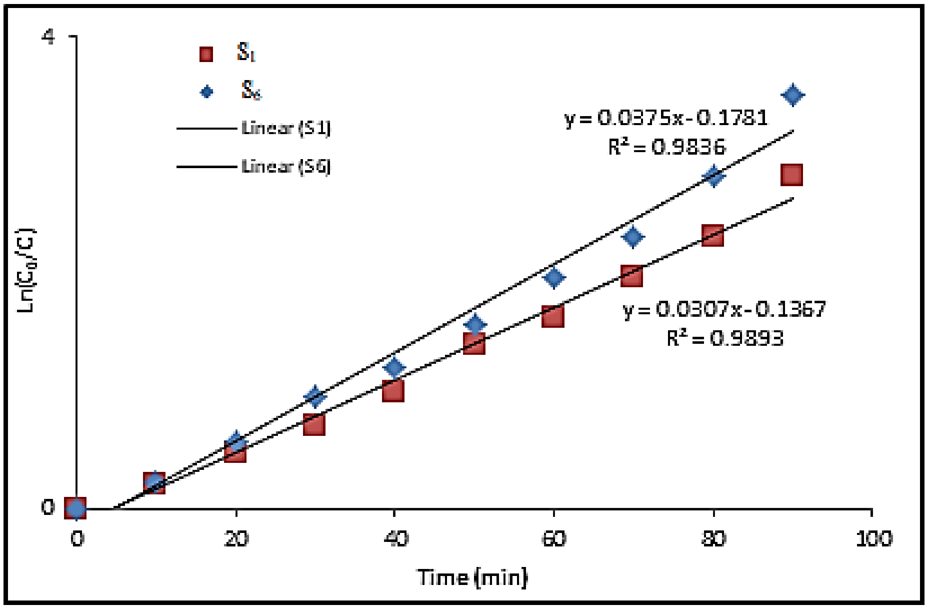

FIGURE S3 ln (C_0/C) versus reaction time plot of the prepared DHPMs

Spectral data for compounds:

5-(Ethoxycarbonyl)-6-methyl-4-phenyl-3,4-dihydropyrimidin-2(1*H*)-one (Table 6, Entry 1): White solid; FT-IR (KBr, *v*, cm^{-1}): 3246 (NH stretch.), 3116 (=C–H stretch.), 2978 (C–H aliphatic stretch.), 1702 (C=O stretch.), 1460, 1422 (C=C aromatic stretch.), 1388 (CH_3 bend.), 1292 (C–N stretch.), 1090 (C–O stretch.); ^{1}H-NMR (DMSO-d_6) δ (ppm): 9.25 (s, 1H), 7.73 (s, 1H), 7.33–7.24 (m, 5H), 5.33 (s, 1H), 3.99 (q, *J* = 6.9 Hz, 2H), 2.26 (s, 3H), 1.09 (t, *J* = 6.9 Hz, 3H).

5-(Ethoxycarbonyl)-6-methyl-4-(4-bromophenyl)-3,4-dihydropyrimidin-2(1*H*)-one (Table 6, Entry 2): White solid; FT-IR (KBr, *v*, cm^{-1}): 3282 (NH stretch.), 3119 (=C–H stretch.), 2932 (C–H aliphatic stretch.), 1704 (C=O stretch.), 1625, 1487 (C=C aromatic stretch.), 1426 (CH_3 bend.), 1229 (C–N stretch.), 1145 (C–O stretch.), 890 (C–Br); ^{1}H-NMR (DMSO-d_6) δ (ppm): 9.34 (s, 1H), 7.82 (s, 1H), 7.57 (d, *J* = 7.1 Hz, 2H), 7.22 (d, *J* = 7.0 Hz, 2H), 5.14 (s, 1H), 4.02 (q, *J* = 6.5 Hz, 2H), 2.28 (s, 3H), 1.19 (t, *J* = 6.5 Hz, 3H).

5-(Ethoxycarbonyl)-6-methyl-4-(2-chlorophenyl)-3,4-dihydropyrimidin-2(1*H*)-one (Table 6, Entry 3): White solid; FT-IR (KBr, *v*, cm^{-1}): 3281 (NH stretch.), 3117 (=C–H stretch.), 2933 (C–H aliphatic stretch.), 1700 (C=O stretch.), 1619 1488 (C=C aromatic stretch.), 1424 (CH_3 bend.), 1230 (C–N stretch.), 1143 (C–O stretch.), 1090 (C–Cl); ^{1}H-NMR (DMSO-d_6) δ (ppm): 9.27 (s, 1H), 7.70 (s, 1H), 7.40 (d, 1H, *J* = 7.7 Hz), 7.41–7.29 (m, 3H), 5.42 (s, 1H), 3.90 (q, *J* = 7.2 Hz, 2H), 2.30 (s, 3H), 0.99 (t, 3H, *J* = 7.1 Hz).

5-(Ethoxycarbonyl)-6-methyl-4-(4-chlorophenyl)-3,4-dihydropyrimidin-2(1*H*)-one (Table 6, Entry 4): White solid; FT-IR (KBr, *v*, cm^{-1}): 3279 (NH stretch.), 3118 (=C–H stretch.), 2931 (C–H aliphatic stretch.), 1701 (C=O stretch.), 1618, 1487 (C=C aromatic stretch.), 1426 (CH_3 bend.), 1234 (C–N stretch.), 1141 (C–O stretch.), 1092 (C–Cl); ^{1}H-NMR (DMSO-d_6) δ (ppm): 9.27 (s, 1H), 7.78 (s, 1H), 7.39 (d, *J* = 8.1 Hz, 2H), 7.26 (d, *J* = 8.1 Hz, 2H), 5.10 (s, 1H), 3.98 (q, *J* = 7.1 Hz, 2H), 2.26 (s, 3H), 1.09 (t, *J* = 7.2 Hz, 3H).

5-(Ethoxycarbonyl)-6-methyl-4-(4-methylphenyl)-3,4-Dihydropyrimidin-2(1*H*)-one (Table 6, Entry 5): White solid; FT-IR (KBr, *v*, cm^{-1}): 3246 (NH stretch.), 3118 (=C–H stretch.), 2930 (C–H aliphatic stretch.), 1704 (C=O stretch.), 1463, 1421 (C=C aromatic stretch.), 1382 (CH_3 bend.), 1290 (C–N stretch.), 1088 (C–O stretch.); ^{1}H-NMR (DMSO-d_6)

δ (ppm): 9.12 (s, 1H), 7.70 (s, 1H), 7.15–7.10 (m, 4H), 5.19 (s, 1H), 3.97 (q, *J* = 7.1 Hz, 2H), 2.25 (s, 3H), 2.24 (s, 3H), 1.10 (t, *J* = 7.1 Hz, 3H)

5-(Ethoxycarbonyl)-6-methyl-4-(4-methoxyphenyl)-3,4-dihydropyrimidin-2(1*H*)-one (Table 6, Entry 6): White solid; FT-IR (KBr, *v*, cm^{-1}): 3238 (NH stretch.), 3120 (=C–H stretch.), 2986 (C–H aliphatic stretch.), 1700 (C=O stretch.), 1469, 1425 (C=C aromatic stretch.), 1383 (CH_3 bend.), 1290 (C–N stretch.), 1088 (C–O stretch.); ^{1}H- NMR (DMSO-d_6) δ (ppm): 9.18 (s, 1H), 7.67 (s, 1H), 7.15 (d, *J* = 8.5 Hz, 2H), 6.87 (d, *J* = 8.2 Hz, 2H), 5.11 (s, 1H), 3.98 (q, *J* = 7.2 Hz, 2H), 3.71 (s, 3H), 2.24 (s, 3H), 1.10 (t, *J* = 7.2 Hz, 3H).

5-(Ethoxycarbonyl)-6-methyl-4-(4-nitrophenyl)-3,4-dihydropyrimidin-2(1*H*)-one (Table 6, Entry 7): White solid; FT-IR (KBr, *v*, cm^{-1}): 3250 (NH stretch.), 3117 (=C–H stretch.), 2980 (C–H aliphatic stretch.), 1706 (C=O stretch.), 1465, 1420 (C=C aromatic stretch.), 1386 (CH_3 bend.), 1293 (C–N stretch.), 1089 (C–O stretch.); ^{1}H-NMR (DMSO-d_6) δ (ppm): 9.37 (s, 1H), 8.21 (d, *J* = 8.2 Hz, 2H), 7.90 (s, 1H), 7.51 (d, *J* = 8.2 Hz, 2H), 5.22 (s, 1H), 3.97 (q, *J* = 7.1 Hz, 2H), 2.27 (s, 3H), 1.09 (t, *J* = 7.1 Hz, 3H).

5-(Ethoxycarbonyl)-6-methyl-4-(2-hydroxyphenyl)-3,4-dihydropyrimidin-2(1*H*)-one (Table 6, Entry 8): White solid; FT-IR (KBr, *v*, cm^{-1}): 3376 (O–H stretch.), 3238 (NH stretch.), 3125 (=C–H stretch.), 2991 (C–H aliphatic stretch.), 1703 (C=O stretch.), 1468, 1420 (C=C aromatic stretch.), 1388 (CH_3 bend.), 1294 (C–N stretch.), 1088 (C–O stretch.); ^{1}H-NMR (DMSO-d_6) δ (ppm): 9.63 (s, 1H), 9.14 (s, 1H), 7.15 (s, 1H), 7.11–7.07 (m, 1H), 7.02 (d, *J* = 7.2 Hz, 1H), 6.83 (d, *J* = 8.0 Hz, 1H), 6.75 (t, *J* = 7.4 Hz, 1H), 5.50 (d, *J* = 2.8 Hz, 1H), 4.00- 3.94 (m, 2H), 2.31 (s, 3H), 1.08 (t, *J* = 7.0 Hz, 3H);

5-(Ethoxycarbonyl)-6-methyl-4-(4-hydroxyphenyl)-3,4-dihydropyrimidin-2(1*H*)-one (Table 6, Entry 9): White solid; FT-IR (KBr, *v*, cm^{-1}): 3377 (O–H stretch.), 3237 (NH stretch.), 3123 (=C–H stretch.), 2990 (C–H aliphatic stretch.), 1701 (C=O stretch.), 1467, 1425 (C=C aromatic stretch.), 1387 (CH_3 bend.), 1292 (C–N stretch.), 1091 (C–O stretch.); ^{1}H-NMR (DMSO-d_6) δ (ppm): 9.30 (s, 1H), 9.11 (s, 1H), 7.66 (s, 1H), 7.06 (d, *J* = 7.8 Hz, 2H), 6.72 (d, *J* = 7.5 Hz, 2H), 5.07 (s, 1H), 4.01 (d, *J* = 6.9 Hz, 2H), 2.26 (s, 3H), 1.13 (t, *J* = 6.9 Hz, 3H).

5-(Methoxycarbonyl)-6-methyl-4-phenyl-3,4-dihydropyrimidin-2(1*H*)-one (Table 6, Entry 10): White solid; FT-IR (KBr, *v*, cm^{-1}): 3242 (NH stretch.), 3125 (=C–H stretch.), 2991 (C–H aliphatic stretch.), 1704 (C=O stretch.), 1469, 1422 (C=C aromatic stretch.), 1389 (CH_3 bend.), 1290 (C–N stretch.), 1092 (C–O stretch.); ^{1}H-NMR (DMSO-d_6) δ (ppm): 9.26 (s, 1H), 7.78 (s, 1H), 7.32 (t, *J* = 7.5 Hz, 2H), 7.27–7.22 (m, 3H), 5.14 (s, 1H), 3.59 (s, 3H), 2.23 (s, 3H).

5-(Methoxycarbonyl)-6-methyl-4-(4-bromophenyl)-3,4-dihydropyrimidin-2(1*H*)-one (Table 6, Entry 11): White solid; FT-IR (KBr, *v*, cm^{-1}): 3251 (NH stretch.), 3122 (=C–H stretch.), 2988 (C–H aliphatic stretch.), 1705 (C=O stretch.), 1471, 1420 (C=C aromatic stretch.), 1389 (CH_3 bend.), 1291 (C–N stretch.), 1094 (C–O stretch.); ^{1}H-NMR (DMSO-d_6) δ (ppm): 9.39 (s, 1H), 7.85 (s, 1H), 7.55 (d, *J* = 8.0 Hz, 2H), 7.22 (d, *J* = 8.1 Hz, 2H), 5.16 (s, 1H), 3.56 (s, 3H), 2.22 (s, 3H),

5-(Methoxycarbonyl)-6-methyl-4-(2-chlorophenyl)-3,4-dihydropyrimidin-2(1*H*)-one (Table 6, Entry 12): White solid; FT-IR (KBr, *v*, cm^{-1}): 3250 (NH stretch.), 3123 (=C–H stretch.), 2987 (C–H aliphatic stretch.), 1701 (C=O stretch.), 1470, 1422 (C=C aromatic stretch.), 1388 (CH_3 bend.), 1294 (C–N stretch.), 1090 (C–O stretch.); ^{1}H-NMR (DMSO-d_6) δ (ppm): 9.28 (s, 1H), 7.63 (s, 1H), 7.42−7.37 (m, 1H), 7.36−7.25 (m, 3H), 5.62 (s, 1H), 3.45 (s, 3H), 2.27 (s, 3H).

5-(Methoxycarbonyl)-6-methyl-4-(4-chlorophenyl)-3,4-dihydropyrimidin-2(1*H*)-one (Table 6, Entry 13): White solid; FT-IR (KBr, *v*, cm^{-1}): 3240 (NH stretch.), 3126 (=C-H stretch.), 2990 (C–H aliphatic stretch.), 1703 (C=O stretch.), 1471, 1423 (C=C aromatic stretch.), 1387 (CH_3 bend.), 1291 (C–N stretch.), 1093 (C–O stretch.); ^{1}H-NMR (DMSO-d_6) δ (ppm): 9.30 (s, 1H), 7.87 (s, 1H), 7.43 (d, *J* = 8.1 Hz, 2H), 7.29 (d, *J* = 8.1 Hz, 2H), 5.19 (s, 1H), 3.59 (s, 3H), 2.34 (s, 3H).

5-(Methoxycarbonyl)-6-methyl-4-(4-methylphenyl)-3,4-dihydropyrimidin-2(1*H*)-one (Table 6, Entry 14): White solid; FT-IR (KBr, *v*, cm^{-1}): 3250 (NH stretch.), 3120 (=C–H stretch.), 2928 (C–H aliphatic stretch.), 1706 (C=O stretch.), 1465, 1422 (C=C aromatic stretch.), 1380 (CH_3 bend.), 1292 (C–N stretch.), 1089 (C–O stretch.); ^{1}H-NMR (DMSO-d_6) δ (ppm): 9.21 (s, 1H), 7.18 (s, 1H), 7.17–7.14 (m, 4H), 5.40 (s, 1H), 3.56 (s, 3H), 2.29 (s, 3H), 2.28 (s, 3H).

5-(Methoxycarbonyl)-6-methyl-4-(4-methoxyphenyl)-3,4-dihydropyrimidin-2(1*H*)-one (Table 6, Entry 15): White solid; FT-IR (KBr, *v*, cm^{-1}): 3245 (NH stretch.), 3120 (=C–H stretch.), 2981 (C–H aliphatic stretch.), 1705 (C=O stretch.), 1466, 1413 (C=C aromatic stretch.), 1387 (CH_3 bend.), 1293 (C–N stretch.), 1089 (C–O stretch.); ^{1}H-NMR (DMSO-d_6) δ (ppm): 9.23 (s, 1H), 7.70 (s, 1H), 7.14 (d, *J* = 7.1 Hz, 2H), 6.88 (d, *J* = 7.1 Hz, 2H), 5.15 (s, 1H,), 3.72 (s, 3H), 3.52 (s, 3H), 2.27 (s, 3H).

5-(Methoxycarbonyl-6-methyl-4-(4-nitrophenyl)-3,4-dihydropyrimidin-2(1*H*)-one (Table 6, Entry 16): White solid; FT-IR (KBr, *v*, cm^{-1}): 3246 (NH stretch.), 3119 (=C–H stretch.), 2983 (C–H aliphatic stretch.), 1702 (C=O stretch.), 1469, 1418 (C=C aromatic stretch.), 1385 (CH_3 bend.), 1290 (C–N stretch.), 1091 (C–O stretch.); ^{1}H-NMR (DMSO-d_6) δ (ppm): 9.34 (s, 1H), 8.21 (d, *J* = 7.0 Hz, 2H), 7.89 (s, 1H), 7.51 (d, *J* = 7.0 Hz, 2H), 5.29 (s, 1H), 3.54 (s, 3H), 2.27 (s, 3H).

5-(Methoxycarbonyl)-6-methyl-4-(2-hydroxyphenyl)-3,4-dihydropyrimidin-2(1*H*)-one (Table 6, Entry 17): White solid; FT-IR (KBr, *v*, cm^{-1}): 3380 (O–H stretch.), 3244 (NH stretch.), 3123 (=C–H stretch.), 2985 (C–H aliphatic stretch.), 1704 (C=O stretch.), 1469, 1420 (C=C aromatic stretch.), 1389 (CH_3 bend.), 1292 (C–N stretch.), 1090 (C-O stretch.); ^{1}H-NMR (DMSO-d_6) δ (ppm): 9.77 (s, 1H), 9.13 (s, 1H), 7.13–6.82 (m, 5H), 5.41 (s, 1H), 3.51 (s, 3H), 2.56 (s, 3H).

5-(Methoxycarbonyl)-6-methyl-4-(4-hydroxyphenyl)-3,4-dihydropyrimidin-2(1*H*)-one (Table 6, Entry 18): White solid; FT-IR (KBr, *v*, cm^{-1}): 3379 (O–H stretch.), 3240 (NH stretch.), 3120 (=C–H stretch.), 2981 (C–H aliphatic stretch.), 1703 (C=O stretch.), 1470, 1423 (C=C aromatic stretch.), 1379 (CH_3 bend.), 1290 (C–N stretch.), 1091 (C–O stretch.); ^{1}H-NMR (DMSO-d_6) δ (ppm): 9.38 (s, 1H), 9.13 (s, 1H), 7.61 (s, 1H), 7.04–7.00 (m, 2H), 6.71–6.65 (m, 2H), 5.24 (s, 1H), 3.52 (s, 3H), 2.30 (s, 3H).

5-(Ethoxycarbonyl)-6-methyl-4-(2-furfuryl)-3,4-dihydropyrimidin-2(1*H*)-one (Table 6, Entry 19): White solid; FT-IR (KBr, *v*, cm^{-1}): 3318 (O–H stretch.), 3226 (NH stretch.), 3105 (=C–H stretch.), 2979 (C–H aliphatic stretch.), 1693 (C=O stretch.), 1471, 1420 (C=C aromatic stretch.), 1378 (CH_3 bend.), 1291 (C–N stretch.), 1092 (C–O stretch.); ^{1}H-NMR (DMSO-d_6) δ (ppm): 9.20 (s, 1H), 7.71 (s, 1H), 7.58 (s, 1H), 6.29 (s, 1H) , 6.11 (s, 1H), 5.08 (s, 1H), 4.00 (q, *J* = 7.0 Hz, 2H), 2.16 (s, 3H), 1.10 (t, *J* = 6.8 Hz, 3H).

5-(Ethoxycarbonyl)-6-methyl-4-(2-thienyl)-3,4-dihydropyrimidin-2(1*H*)-one (Table 6, Entry 20): White solid; FT-IR (KBr, *v*, cm^{-1}): 3312 (O–H stretch.), 3219 (NH stretch.), 3109 (=C–H stretch.), 2975 (C–H aliphatic stretch.), 1690 (C=O stretch.), 1472, 1424 (C=C aromatic stretch.), 1372 (CH_3 bend.), 1290 (C–N stretch.), 1094 (C–O stretch.); ^{1}H-NMR (DMSO-d_6) δ (ppm): 9.28 (s, 1H), 7.80 (s, 1H), 7.39 (d, *J* = 6.0 Hz, 1H), 6.94-6.80 (m, 2H), 5.31 (s, 1H), 4.07 (q, *J* = 6.9 Hz, 2H), 2.23 (s, 3H), 1.19 (t, *J* = 7.0 Hz, 3H).
